# Presentations to an urban emergency department in Switzerland due to acute γ-hydroxybutyrate toxicity

**DOI:** 10.1186/s13049-016-0299-z

**Published:** 2016-08-31

**Authors:** Evangelia Liakoni, Fabio Walther, Christian H. Nickel, Matthias E. Liechti

**Affiliations:** 1 Division of Clinical Pharmacology and Toxicology, University Hospital Basel and University of Basel, Basel, Switzerland; 2 Emergency Department, University Hospital Basel and University of Basel, Basel, Switzerland

**Keywords:** GHB, Acute toxicity, Gamma-hydroxybutyrate

## Abstract

**Background:**

γ-Hydroxybutyrate (GHB) is a drug of abuse with dose-dependent sedative effects. Systematic data on the acute toxicity of GHB from emergency department (ED) presentations over a long period of time are currently missing from the literature. The present study described the clinical features of GHB toxicity.

**Methods:**

Retrospective case series of GHB intoxications seen in an urban ED.

**Results:**

From January 2002 to September 2015, 78 GHB-related intoxication cases were recorded (71 % male patients). The mean ± SD age was 29 ± 8 years. The co-use of alcohol and/or other illicit drugs was reported in 65 % of the cases. Neurological symptoms other than central nervous system depression included agitation (40 %) and clonus (21 %). The most frequent reasons for admission were coma (64 %) and agitation (23 %). The median time to regain consciousness was 90 min (range, 3–400 min). Sudden recovery was reported in 25 cases (32 %). Coma was not significantly associated with polyintoxication. Coma occurred in 77 % of the alcohol co-users and in 62 % ofthe non-alcohol users (*p*=0.052). The mean recovery time in comatose patients was 142 min in patients with co-use of alcohol compared with 89 min in patients without alcohol co-use (*p*=0.07). Alcohol co-use was not significantly associated with nausea/vomiting (*p*=0.07). The co-use of stimulants was not significantly associated with non-responsive coma (Glasgow Coma Scale = 3) or mean recovery time. Analytical confirmation of GHB was available in 37 cases (47 %), with additional quantitative analysis in 20 cases. The median GHB concentration was 240 mg/L (range, 8.3–373 mg/L). Intoxication was severe in 72 % of the cases. No fatalities occurred, and 72 % of the patients were discharged directly home from the ED.

**Discussion:**

There were trend associations between alcohol co-use and frequency and length of coma and nausea/vomiting which did not reach the significance level (all *p*=0.05-0.07) but may nevertheless be clinically relevant. As the exact time of use is not always known, and co-use of other substances can affect the severity of poisoning, no definitive conclusions can be drawn regarding the association between GHB concentration and severity.

**Conclusion:**

Impaired consciousness and agitation were typical findings of GHB intoxication. The co-use of alcohol and/or other illicit substances is common but was not significantly associated with the severity of the intoxications in our study.

## Background

γ-Hydroxybutyrate (GHB), commonly known as “liquid ecstasy,” and its precursors γ-butyrolactone (GBL) and 1,4-butanediol (BD) emerged as drugs of abuse in the early 1990s. Since that time, they have led to a greater burden on healthcare providers [1–3]. After ingestion, GBL and BD are rapidly converted to GHB, thereby producing the same clinical effects [1, 3]. Throughout the present article, reference to GHB implicitly includes GBL and BD. GHB stimulates GHB and γ-aminobutyric acid (GABA) receptors [4]. At lower doses, GHB produces mixed stimulant/sedative effects, with a dose-dependent increase in sedation and dizziness [5]. At higher doses, GHB can lead to severe coma, cardiorespiratory depression, and death [1, 2, 6, 7]. There is a non-linear dose-plasma concentration relationship, with higher doses producing disproportionately higher plasma exposure and a potentially high risk of overdose [5, 8, 9]. The plasma elimination half-life of GHB is short (20–50 min), leading to a short time window of detection (i.e., ≤ 4-5 h in blood, ≤ 12 h in urine) [1, 2, 5, 10, 11].

Currently, only relatively limited systematic and detailed data are available on the acute toxicity of GHB that have been collected in a standardized manner based on emergency department (ED) presentations. The European Monitoring Centre for Drugs and Drug Addiction (EMCDDA) [12] collects and reports annually data on the prevalence of drug use, including GHB. However, prevalence data do not provide information on the acute toxicity of these substances. Surveys among GHB users [13, 14] revealed that overdoses are common, especially among new users. The most frequently reported reasons for use are recreation and sexual enhancement. According to the European Drug Emergency Network (Euro-DEN), which collected data on acute drug toxicity from 16 EDs in 10 European countries, GHB was among the top five drugs recorded [15]. Data have also been collected from medical stations during “rave” parties to investigate the time course of awakening from GHB intoxication and the relationship to plasma concentrations [16]. This case series illustrated that patients with GHB intoxication had a Glasgow Coma Scale (GCS) score ≤ 8 for a median time of 90 min (range, 30–105 min), followed by sudden recovery over ~30 min (range, 10–50 min). The median GHB plasma concentration upon arrival was 212 μg/ml (range, 112–430 μg/ml), and awakening was accompanied by a small change in GHB concentrations. However, a confounding factor was the co-use of other illicit drugs in 14 of the 15 cases. A retrospective study of fatal GHB-related cases analyzed the cause of death; however, in this study, 21 of the 23 cases (91 %) had co-used other substances [17]. Data from patients who presented to EDs with acute GHB toxicity are also available [6, 7, 15, 18–24]. According to these studies, GHB intoxication frequently results in coma, with typically rapid spontaneous recovery. The typical patient is young and male, and the co-use of alcohol and/or other illicit substances is common. In terms of management, some authors (e.g., [24]) have argued that conservative management might be preferable to intubation. In one study, agitation was more frequent when alcohol was co-used. In contrast to what might be expected, the co-use of cocaine and methylenedioxymethamphetamine (MDMA; ecstasy) induced more pronounced and prolonged coma [6]. A possible explanation for this observation could be that higher amounts of GHB were used in these cases to “calm down” [6]. GHB intoxication is associated with substantial morbidity [21].

However, systematic data over a longer period of time are missing from the literature. Therefore, the present study sought to describe the characteristics of GHB toxicity that resulted in presentation to a large urban ED in Switzerland over a 14-year period (2002–2015). The possible moderation of GHB toxicity by the co-use of other substances was also investigated. Specifically, we examined possible associations between the co-use of other illicit substances and/or alcohol and the severity of poisoning, coma, agitation, the number of intubations, intensive care unit (ICU) admission, and nausea/vomiting. We also examined possible associations between the co-use of stimulants and the depth and duration of coma.

## Methods

The study was approved by the ethics committee of northwestern Switzerland (no. 163/08). We included all cases that were related to GHB use who presented to the ED of the University Hospital of Basel between January 1, 2002, and September 30, 2015. The University Hospital Basel is one of Switzerland's leading university medical centers, serving as a primary care and referral center for northwestern Switzerland. All emergency patients (50,000/year) are first evaluated by the ED.

Cases were retrieved from the electronic patient chart database using a comprehensive full-text search algorithm [25]. Briefly, the sensitive automatic search identified all cases that mentioned GHB, GBL, or related terms (e.g., abbreviations and misspellings). Data abstraction was performed by two of the authors of the study. The complete patient charts of all of the retrieved cases were reviewed, including notes by paramedics. All cases that were related to GHB toxicity were included in the study. GHB use was determined based on the patients’ self-reported use and/or analytical confirmation. One case with typical clinical findings and a bottle of GHB that was found in the patient’s pocket was also included.

A retrospective study design was used with standardized data recording [6, 15, 26]. Using full patient charts, we recorded age, sex, hour and day of the ED visit, heart rate, diastolic and systolic blood pressure, GCS score, body temperature, and laboratory test results. We also recorded any co-ingestion of alcohol or other drugs, both based on self-report and/or the results of blood and urine toxicology tests (when available). Coma was defined as GCS = 3-8 and/or documented coma. “Non-responsive coma” was defined as GCS = 3. In comatose patients, recovery time was defined as the time from initial presentation to paramedics or the ED until the patient awoke, confirmed by a note in the chart or GCS = 14 or 15. The term “polyintoxication” refers to the reported co-use of other drugs and/or alcohol, except as otherwise noted. The severity of poisoning was assessed using the Poison Severity Score to grade acute poisoning [27]. Mild toxicity refers to mild, transient, and spontaneously resolving symptoms. Moderate toxicity refers to pronounced or prolonged symptoms. Severe toxicity refers to severe or life-threatening symptoms [27].

Levels of GHB were determined using gas chromatography–mass spectrometry and an enzymatic test assay (limit of detection = 1.5 mg/L, limit of quantification = 5 mg/L). Group differences were analyzed using *χ*^*2*^ tests (proportions) or nonparametric Mann-Whitney U tests (mean ranks). Spearman rank correlations were used to assess associations between variables. The data were analyzed using Statistica 12 software (StatSoft, Tulsa, OK, USA). Values of *p* < 0.05 were considered statistically significant.

## Results

### Number of admissions and patient characteristics

Over the 14 years of the study, from 2002 to 2015, we recorded 78 GHB-related intoxication cases in 60 different patients. Six patients presented more than once to the ED (two patients twice, one patient three times, one patient four times, one patient six times, and one patient seven times). The patient characteristics are shown in Table 1.

**Table 1:** Patient characteristics

	Intoxications (*n* = 78) (%)
Age (years)	
17-20	13 (17)
21-25	15 (19)
26-30	17 (22)
31-35	13 (17)
36-40	16 (21)
41-50	4 (5)
Prior history of illicit drug use	50 (64)
GHB	41 (53)
Cannabis	16 (21)
Opiates	13 (17)
Cocaine	14 (18)
MDMA (ecstasy)/other amphetamines	13 (17)
Time of presentation (arrival)	
Night (8:00 PM–8:00 AM)	41 (53)
Weekend (Friday 5:00 PM–Monday 8:00 AM)	36 (46)
Monday–Friday (8:00 AM–8:00 PM)	24 (31)
Saturday–Sunday (8:00 AM–8:00 PM)	8 (10)
Monday–Thursday (8:00 PM–8:00 AM)	14 (18)
Friday–Sunday (8:00 PM–8:00 AM)	30 (38)
Weekday, time of arrival not recorded	2 (3)
Context of use	
Recreational substance use	70 (90)
Accidental ingestion	3 (4)
Poisoning	3 (4)
Suicide attempt	2 (3)
Brought to emergency department by	
Ambulance	62 (79)
Ambulance transport from a psychiatric ward	5 (6)
Police	2 (3)
Other (friends, partner, etc.)	6 (8)
Unknown/not reported	3 (4)

**Fig. 1:**
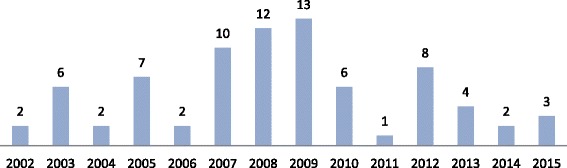
Annual incidence of cases of GHB intoxication

The mean ± SD age was 29.0 ± 8.0 years, and most of the patients (71 %) were male. The majority of the patients (64 %) had a documented and/or self-reported prior history of illicit substance use, including GHB in 41 cases (53 %). Figure 1 shows the cases according to year.

### Clinical findings

The clinical signs and symptoms of GHB toxicity are shown in Table 2.

**Table 2:** Characteristics of GHB intoxication

	Intoxications (*n* = 78) (%)
Cause of admission	
Coma (GCS = 3-8 and/or documented coma)	50 (64)
Agitation/aggression	18 (23)
Collapse/syncope	7 (9)
Convulsions/seizures	6 (8)
Confusion	2 (3)
Dizziness	2 (3)
Anxiety	1 (1)
Nausea	1 (1)
Delirium	1 (1)
Suspected intoxication	1 (1)
Hyperventilation	1 (1)
GHB identification	
Self-report	40 (51)
Toxicological analysis	24 (31)
Self-report and toxicological analysis	13 (17)
GHB bottle in pocket	1 (1)
Initial Glasgow Coma Scale score	
3	13 (17)
4-8	16 (21)
9-12	17 (22)
13-15	22 (28)
Unknown	10 (13)
Lowest Glasgow Coma Scale score	
3	18 (23)
4-8	17 (22)
9-12	11 (14)
13-15	20 (26)
Unknown	12 (15)
Clinical findings any time prior or during presentation to the emergency department	
Coma (GCS = 3-8 and/or documented coma)	53 (68)
Agitation	31 (40)
Hypotension (systolic blood pressure < 95 mmHg)	19 (24)
Clonus	16 (21)
Bradycardia (<60 beats/min)	12 (15)
Hypothermia (< 36 °C)	7 (9)
Respiratory insufficiency (oxygen saturation < 92 %)	7 (9)
Nausea/vomiting	7 (9)
Urine or stool incontinence	4 (5)
Management	
Monitoring	64 (82)
Intravenous fluids	32 (41)
Oxygen administration	24 (31)
Intubation	8 (10)
Urinary catheterization	3 (4)
External heating	2 (3)
Medication	
Benzodiazepines	10 (13)
Naloxone	8 (10)
Flumazenil	1 (1)
Severity of poisoning (based on Poison Severity Score [27])	
Minor	5 (6)
Moderate	17 (22)
Severe	56 (72)
Discharge from emergency department	
To home	56 (72)
To intensive care unit	13 (17)
To psychiatric clinic	9 (12)

**Fig. 2:**
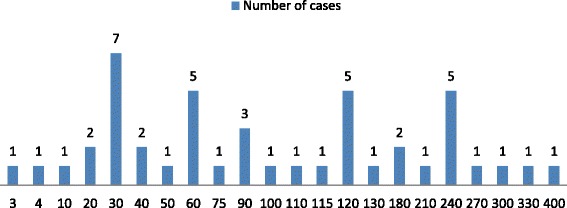
Time to regain consciousness (in minutes)

The most common cause for admission was coma (50 cases [64 %]), followed by agitation (18 cases [23 %]). A GCS score of 3, indicating non-reactive coma, was recorded in 13 cases (17 %) at initial presentation and in 18 cases (23 %) at any time during observation. The initial GCS score negatively correlated with time to recovery (*R*_*s*_ = -0.53, *p* < 0.001). Sudden recovery was reported in 25 cases (32 %). The median time to regain consciousness was 90 min (range, 3–400 min; Fig. 2).

Neurological symptoms other than central nervous system depression included agitation (31 cases [40 %]) and clonus (16 cases [21 %]). An electrocardiogram was performed in 27 cases (35 %), the significant findings of which were sinus bradycardia in three cases, slow R-progression in one case, ST-elevation in one case, and T-wave elevation in one case. Computed tomography of the head was performed in 18 cases (23 %), without significant findings. Psychiatric assessment took place in 18 cases (23 %), and nine patients (12 %) had to be transferred to a psychiatric clinic (half of them according to the psychiatric assessment and the other half without involvement of a psychiatrist). Among the patients with a GCS score of 3-8 and/or documented coma any time during the observation (a total of 53 cases), 12 (23 %) were admitted to the ICU and eight (15 %) were intubated; computed tomography of the head was performed in 14 (26 %) of these cases.

### Use of other substances

In 36 cases (46 %), other illicit drugs were used in combination with GHB according to the patients’ self-reports (Table 3).

**Table 3:** Concomitant substance use

	Intoxications (*n* = 78) (%)
Concomitant substance use	
GHB monointoxication (no other illicit drugs or alcohol reported)	27 (35)
GHB polyintoxication (reported co-use of other drugs and/or alcohol)	51 (65)
Reported co-use of alcohol	26 (33)
Reported and/or laboratory-confirmed co-use of alcohol	31 (40)
Co-use of other illicit drugs (according to patient self-report)	36 (46)
Cannabis	14 (18)
Cocaine	13 (17)
Sedatives	12 (15)
Opiates	8 (10)
Amphetamine	4 (5)
MDMA (ecstasy)	4 (5)
Ketamine	1 (1)
Laboratory test data	70 (90)
Ethanol test performed	66 (85)
Positive ethanol test	23 (29)
Drug test performed	67 (86)
Positive for benzodiazepines	15 (19)
Positive for cannabis	13 (17)
Positive for cocaine	10 (13)
Positive for methadone	4 (5)
Positive for opiates	2 (3)
Positive for amphetamines	1 (1)

An ethanol test was performed in 66 cases (85 %). Among these cases, a negative result was recorded in 43 cases. Among the 23 cases with positive ethanol tests, the median alcohol concentration was 0.8 g/L (range, 0.1–2.9 g/L). The toxicological test results for substances other than GHB are shown in Table 3. Among the GHB monointoxication cases (i.e., no other illicit drugs or alcohol reported, *n* = 27), toxicological drug screening test results were available in 23 cases. Among these 23 cases, 20 were negative and three were positive for benzodiazepines. An ethanol test was performed in all 20 of these cases, with no self-reported co-use of other illicit drugs or alcohol and a negative toxicological drug screening test. The ethanol test was negative in 18 of the cases and positive in two of the cases. Among the cases with self-reported co-use of other illicit drugs (*n* = 36), toxicological drug screening test results were available in 31 cases. Among these, five were negative and 26 were positive. Among the positive tests, the same substances as those that were self-reported were detected in 21 cases. More substances than those that were self-reported were detected with drug tests in four cases, and fewer substances than those that were self-reported were detected in one case.

### Cases with analytical GHB confirmation

Confirmation of GHB was available in 37 cases (47 %), and a quantitative analysis of GHB was available in 20 cases (26 %). The median concentration was 240 mg/L (range, 8.3–373 mg/L). The characteristics of the 20 intoxications with quantitative analysis of GHB are shown in Table 4.

**Table 4:** Characteristics of GHB intoxications with quantitative analysis of GHB (*n* = 20)

Gender	Age	Other substances reported and/or analytically confirmed	Severity of poisoning	GHB concentration in blood
Male	37	Benzodiazepines, cocaine	Moderate	8.3 mg/L
Male	23	Cannabis, cocaine	Severe	309 mg/L
Female	19	Alcohol, cannabis	Severe	31 mg/L
Male	20	Alcohol	Moderate	29 mg/L
Female	22	Alcohol	Severe	95 mg/L
Male	35	Alcohol	Severe	59 mg/L
Male	47	Alcohol, cannabis, methadone	Severe	83 mg/L
Female	19	—	Severe	259 mg/L (82 mg/L in urine)
Female	17	—	Moderate	62 mg/L
Male	39	Benzodiazepines	Severe	331 mg/L
Female	25	—	Minor	108 mg/L
Male	39	—	Severe	327 mg/L
Male	39	—	Severe	360 mg/L
Male	27	Opiates	Minor	> 20 mg/L
Male	27	—	Moderate	373 mg/L
Male	17	—	Severe	301 mg/L
Male	21	Alcohol, cannabis	Severe	86 mg/L
Female	29	Alcohol, cocaine	Severe	313 mg/L
Male	25	Alcohol, cocaine	Severe	307 mg/L
Female	24	Cocaine	Severe	240 mg/L

### Explanatory causes of severe intoxication

Coma was not significantly associated with polyintoxication. Coma occurred in 75 % (38/51) of the cases with polyintoxication and 56 % (15/27) of the cases with monointoxication (*χ*^*2*^ = 2.9, *p* = 0.09). Non-responsive coma (GCS = 3) was present in 24 % (12/51) of the polyintoxications and 22 % (6/27) of the monointoxications (*χ*^*2*^ = 0.02, *p* = 0.9). Coma occurred in 77 % (24/31) of the alcohol co-users and 62 % (29/47) of the non-alcohol users (*χ*^*2*^ = 3.8, *p* = 0.052). A GCS score of 3 was present in 35 % (11/31) of the alcohol co-users and 38 % (18/47) of the non-alcohol co-users (*χ*^*2*^ = 0.06, *p* = 0.8). The mean recovery time in comatose patients (GCS = 3-8 and/or documented coma) was 142 min (*n* = 22; range, 20–400 min) in patients with co-use of alcohol compared with 89 min (*n* = 23; range, 3–300 min) in patients without alcohol co-use (*Z* = 1.8, *p* = 0.07). Severely intoxicated patients used other substances (alcohol and/or other illicit drugs) in addition to GHB (polyintoxications) in 70 % (39/56) of the cases, and non-severely intoxicated patients used additional substances in 55 % (12/22) of the cases (*χ*^*2*^ = 1.6, *p* = 0.2). The co-use of alcohol (self-reported and/or laboratory-detected; *n* = 31) was documented in 46 % (26/56) of the severely intoxicated patients and 23 % (5/22) of the non-severely intoxicated patients (*χ*^*2*^ = 3.7, *p* = 0.054). Agitation was not more frequent among patients with poly- vs. monointoxications or in patients with or without the co-use of alcohol. Polyintoxications or alcohol co-use were not associated with the number of intubations or ICU admissions. Reported and/or laboratory-confirmed alcohol co-use was not significantly associated with nausea/vomiting (5/31 vs. 2/47; *χ*^*2*^ = 3.2, *p* = 0.07). The co-use of stimulants (e.g., cocaine, amphetamine, and MDMA) was not significantly associated with non-responsive coma (GCS = 3; 3/15 vs. 15/51, *χ*^*2*^ = 0.5, *p* = 0.5) or mean recovery time (*n* = 11; 93 min [range, 10–270 min] vs. 126 min [range, 3–400 min]; *N* = 30, *Z* = 0.77, *p* = 0.4).

## Discussion

This retrospective study described the characteristics of GHB toxicity in patients who presented to a large ED in Switzerland. The typical patient was approximately 30 years old and male, had a history of previous illicit substance use, currently used GHB for recreational purposes (often in combination with alcohol [one-third of cases] and/or other recreational substances [nearly half of cases], most commonly cannabis and cocaine), and was brought to the ED by ambulance at night. The most common causes of admission were coma and agitation. Although impaired consciousness led to characterization of the intoxication as severe in the majority of the cases, sudden recovery was recorded in one-third of the cases, and the majority of the patients were discharged home directly from the ED. No significant association was found between polyintoxication and coma, between the co-use of alcohol and the severity of poisoning, or between the co-use of alcohol or other illicit substances and agitation, intubation, degree of coma, or ICU admission. However, nearly significant (all *p* = 0.05–0.07) associations were found between alcohol co-use and coma frequency, coma duration, nausea/vomiting, and overall severity of intoxication, which were likely clinically relevant. In both the present study and a previous retrospective case-study from Zurich, Switzerland [6], alcohol co-use was not significantly associated with the level of consciousness (i.e., decreased GCS score). In contrast to the study from Zurich, however, we found no significant association between the co-use of alcohol and agitation or nausea/vomiting [6]. We also could not confirm the previous finding of deeper (i.e., non-responsive) and more prolonged coma in co-users of stimulant drugs [6].

Other retrospective case-series with data from ED patients [7, 15, 19, 20, 23] also confirmed the characteristics of typical GHB intoxication, particularly the co-use of alcohol and/or other illicit substances with typically rapid spontaneous recovery (usually within ~2 h of presentation; maximum of 6.5 h after presentation). Because of this rapid recovery, some authors have indicated that intubation may not always be necessary in cases of GHB-induced coma [7]. In more recent studies [24], conservative airway management (i.e., no intubation) appears to be preferable in many cases because intubation is associated with a longer stay in the ED. In the present study, only 15 % of the patients with coma were intubated, and only ~25 % were admitted to the ICU and/or underwent computed tomography of the head. However, because of the frequent co-use of other substances and because the analytical detection of GHB is not routinely performed, general recommendations regarding the need for intubation should be made with caution. In a study from Spain [19], polyintoxication was associated with more severe symptoms. In the same study, a decreasing trend in the number of intoxications was observed in the last 3 years of the study (i.e., 2005–2007). In the present study, most of the GHB-related admissions were recorded between 2007 and 2009. Although no fatalities were reported in our study and in most of the previous ED studies [6, 7, 19, 20, 23], fatalities were reported in case series from Sweden (e.g., a young female patient with severe brain anoxia, deeply comatose on arrival) [21] and the United Kingdom (e.g., 25-year-old male with hypoxic brain injury and cardiorespiratory arrest on arrival, with the cause of death listed as “mixed drug overdose [MDMA and GHB toxicity]”) [22]. However, deaths that may have occurred before admission to the ED of our hospital cannot be excluded.

Antidotes that are available for other substance groups (e.g., naloxone and flumazenil) have no beneficial effect in the treatment of GHB intoxication. For example, the duration of coma does not significantly vary with the use of such antidotes [1, 19]. Currently, the management of GHB toxicity is primarily supportive. However, benzodiazepines appear to be a therapeutic option in cases of GHB withdrawal symptoms (e.g., anxiety, insomnia, tremor, tachycardia, agitation, delirium, and hallucinations), which may occur after long-term abuse, beginning within 1–6 h after taking the last dose [1]. Barbiturates (e.g., phenobarbital), baclofen, or propofol may also be considered treatments, particularly in cases of benzodiazepine-refractory GHB withdrawal [2, 28–30]. Specifically for non-responsive hallucinations, antipsychotics may also be indicated [30].

One concern is the use of GHB to “spike” a person’s drink to engage in non-consensual sexual activity or facilitate other criminal actions, such as robbery [31, 32]. In our study, use for recreational purposes was reported in the large majority of cases (90 %), but three cases were reported as “poisoning,” in which GHB may have been given to facilitate sexual assault. Three cases were recorded as “accidental use,” in which the patients reported not knowing what substance they consumed (n.b., GHB was analytically confirmed). Some of the cases might have been related to abuse but not reported as such. However, accidental ingestion, sometimes lethal, has been reported, including cases of mistaking GHB for water or accidental ingestion by children [33].

One strength of our study is the quantitative analysis of GHB in blood, which was available in one-fourth of the cases, and qualitative substance confirmation, which was available in nearly half of the cases. Previously published series were mainly based only on self-reported GHB use. With a qualitative analysis that was available in 37 cases and additional quantitative analysis that was available in 20 cases, our study may be considered one of the relatively large compilations of analytically proven nonfatal cases. Controlled administration of oral psychoactive doses of GHB of 20-72 mg/kg, which did not produce loss of consciousness, resulted in average maximal plasma levels of 22-130 mg/L [5, 34]. Toxicological analysis in nonfatal cases showed a mean GHB concentration of 245 mg/L (range, 86–551 mg/L; *n* = 20) in one small study [35], a median GHB concentration of 180 mg/L (range, 45–295 mg/L; *n* = 15) in another study [36], and a mean GHB concentration of 137 mg/L (median, 103 mg/L; range, 29–490 mg/L; *n* = 54) in a larger study [18]. Published reports of GHB-related fatalities show a wide range of concentrations that are measured in postmortem blood (0-6500 mg/L) [17, 33, 37, 38], with a median concentration of 190–347 mg/L [17, 33, 37], mean ± SD concentration of 482 ± 758 mg/L [38], and concentration of 300 mg/L that is suggested to be sufficient to cause death [37]. Thus, the concentrations of GHB in survivors and non-survivors seem to overlap. However, the possible production and redistribution of GHB after death (depending on the methodology, concentrations up to 197 mg/L even in cases without GHB ingestion [38]) and several other factors (e.g., elapsed time from ingestion to death and from death to sample collection and testing; methods of sample storage and analysis; different levels based on sampling site; the occurrence of delayed death at low GHB concentrations because of loss of consciousness, hypoventilation, and hypoxia [1, 33, 38]; and the possibility that other substances are the cause of death in polyintoxication cases) make the interpretation of postmortem GHB concentrations difficult, with no clearly defined “lethal” concentration [33]. The measured concentrations in our study (median, 240 mg/L; range, 8.3–373 mg/L) were similar to those reported in the other non-fatal GHB intoxication cases. Blood concentrations > 300 mg/L appear to be associated with severe intoxication (Table 4). However, the co-use of other substances can affect symptoms and thus the severity of poisoning. Even in cases of monointoxication, the exact time of use is not always known, and no definitive conclusions can be drawn regarding the association between GHB concentration and severity. This may also explain the cases of severe intoxication with only low GHB concentrations detected.

Our study has limitations. Although the full-text search of the electronic patient charts likely allowed the retrieval of all cases, cases of GHB use but with no self-reported use by the patient and no physician-suspected substance use may not have been recognized and therefore not included in the study. Moreover, polyintoxication was common, and some of the reported signs and symptoms may be attributable to the use of substances other than GHB. Nevertheless, these limitations are common in all retrospective studies that investigate the acute toxicity of psychoactive substances. The short half-life of elimination of GHB may also have led to undetected levels despite laboratory analysis in some cases, whereas substances that are taken as co-medication or given as treatments by paramedics (e.g., benzodiazepines) or can be detected in samples beyond acute intoxication (e.g., cannabis) may be overrepresented in the analytical results. Furthermore, some data were missing in the patient histories. Clinical data were not always recorded in a standardized manner at presentation, suggesting the possibility of reporting bias (e.g., sudden recovery that was reported in 32 % of the cases may be underrepresented because some clinicians may not have mentioned this in the clinical records). Furthermore, analytical confirmation was not available in all of the cases. Lastly, data from only one ED may not be representative because it may reflect only local trends.

## Conclusion

In conclusion, GHB intoxication resulted in impaired consciousness as a typical finding, with rapid spontaneous recovery in many cases. The co-use of ethanol and/or other illicit substances was common. Future studies that quantitatively analyze more GHB monointoxication cases could help elucidate the relationship between clinical presentation and laboratory results and also define a crucial concentration limit.

## Abbreviations

ED: Emergency department
GABA: γ-aminobutyric acid
GBL: γ-butyrolactone
GCS: Glasgow coma scale
GHB: γ-hydroxybutyrate
